# SIRT1 Inhibition Affects Angiogenic Properties of Human MSCs

**DOI:** 10.1155/2014/783459

**Published:** 2014-08-27

**Authors:** Botti Chiara, Caiafa Ilaria, Coppola Antonietta, Cuomo Francesca, Miceli Marco, Altucci Lucia, Cobellis Gilda

**Affiliations:** ^1^Department of Biochemistry, Biophysics and General Pathology, Second University of Napoli, Via L. De Crecchio 7, 80138 Napoli, Italy; ^2^Istituto Nazionale Tumori, Struttura Complessa Oncologia Medica Melanoma Immunoterapia Oncologica e Terapia Innovativa, Via Mariano Semmola, 80131 Napoli, Italy; ^3^Institute of Genetics and Biophysics “A. Buzzati-Traverso”, CNR, Via P. Castellino 111, 80131 Napoli, Italy

## Abstract

Human mesenchymal stem cells (hMSCs) are attractive for clinical and experimental purposes due to their capability of self-renewal and of differentiating into several cell types. Autologous hMSCs transplantation has been proven to induce therapeutic angiogenesis in ischemic disorders. However, the molecular mechanisms underlying these effects remain unclear. A recent report has connected MSCs multipotency to sirtuin families, showing that SIRT1 can regulate MSCs function. Furthermore, SIRT1 is a critical modulator of endothelial angiogenic functions. Here, we described the generation of an immortalized human mesenchymal bone marrow-derived cell line and we investigated the angiogenic phenotype of our cellular model by inhibiting SIRT1 by both the genetic and pharmacological level. We first assessed the expression of SIRT1 in hMSCs under basal and hypoxic conditions at both RNA and protein level. Inhibition of SIRT1 by sirtinol, a cell-permeable inhibitor, or by specific sh-RNA resulted in an increase of premature-senescence phenotype, a reduction of proliferation rate with increased apoptosis. Furthermore, we observed a consistent reduction of tubule-like formation and migration and we found that SIRT1 inhibition reduced the hypoxia induced accumulation of HIF-1*α* protein and its transcriptional activity in hMSCs. Our findings identify SIRT1 as regulator of hypoxia-induced response in hMSCs and may contribute to the development of new therapeutic strategies to improve regenerative properties of mesenchymal stem cells in ischemic disorders through SIRT1 modulation.

## 1. Introduction

Human mesenchymal stem cells (hMSCs) have become an important tool for cell-based strategies. They can differentiate into a variety of cell types such as muscle, neural precursors, cardiomyocytes, and perivascular cells and are currently being tested in several approved clinical trials [1]. hMSCs can improve myocardial remodeling in infarcted heart [2] or promote angiogenesis in critical limb ischemia [3], due to their capacity to stimulate endothelial progenitor cells. Furthermore, hMSCs support neoangiogenesis also by releasing soluble factors that stimulate angiogenesis [4–9]. However, the molecular mechanisms of beneficial effects from hMSCs-based therapy remain unclear.

A recent report showed that SIRT1 might regulate MSCs function, providing a connection between sirtuin families and MSCs multipotency [10].

Sirtuins are classified as class III histone deacetylases (HDACs) [11], originally identified in yeast. They modulate a wide range of biological processes, spanning from DNA repair and oxidative stress responses to energy metabolism. Sirtuins activity is controlled by the cellular [NAD+]/[NADH] ratio, where NAD+ works as an activator, whereas nicotinamide and NADH act as inhibitors.

In mammals, the sirtuin family comprises seven members (SIRT1–SIRT7) with different biological functions and subcellular localizations [12–14]. SIRT1, SIRT6, and SIRT7 are mainly nuclear, whereas SIRT2 is found primarily in the cytosol. SIRT3, SIRT4, and SIRT5 are mitochondrial proteins [12]. Sirtuins are generally known to regulate the acetylation levels and the activity of histone and nonhistone regulatory proteins.

To date, sirtuins have emerged as potential therapeutic targets for treatment of human pathologies such as cardiovascular disease, inflammation, and cancer [15]. SIRT1 is the most studied member of sirtuins. It acts in various cellular processes and exerts its action activating and deactivating factors such as NF-*κ*B, p53, p73, SOD, and hypoxia-inducible transcription factors (HIFs) [16–19].

Since SIRT1 targets several proteins in distinct signaling pathways, modulation of SIRT1 activity alters the biological activity of entire signaling networks modifying disease progression, such as pathological angiogenesis or atherosclerosis.

An important component of pathological angiogenesis is represented by hypoxia that alters the cellular redox state and activates SIRT1. In addition, hypoxia works through multiple pathways to regulate angiogenesis, for instance, through the modulation of secreted angiogenic proteins, such as vascular endothelial growth factor (VEGF), stimulated by increased expression of transcription factors such as HIF-1*α* [20].

Furthermore, a “protective” role of SIRT1 in endothelial cells was described [21, 22]. A recent report has investigated the function of SIRT1 in regulating the differentiation of mesenchymal stem cells by deacetylating *β*-catenin in mice [10]. Several studies demonstrated that inhibition of SIRT1 impairs cell growth in cancer cells [23, 24]. Gorenne et al. [25] reported that SIRT1 expression was reduced in human atherosclerotic plaques and in vascular smooth muscle cells. However, to the best of our knowledge, the effects of SIRT1 modulation on angiogenesis in hMSCs have not been studied yet.

In this study, we described the generation of an immortalized human mesenchymal bone marrow-derived cell line (MeBM) and we investigated whether SIRT1 has an effect on angiogenic capability of hMSCs by inhibiting SIRT1 through pharmacological and genetic approaches.

Since recent studies have identified SIRT1 as a critical modulator of angiogenesis [18, 26], we tested whether the inhibition of SIRT1 activity is associated with the reduction of angiogenic ability of hMSCs and impaired hypoxic response in these settings. We found that the inhibition of SIRT1 activity resulted in reduced capacity to proliferate, to migrate, and to form three-dimensional networks of vessel-like structures. In addition, SIRT1 inhibition reduced the hypoxia-induced accumulation of HIF-1*α* and its transcriptional activity in hMSCs.

Our results suggested that SIRT1 is involved in angiogenic response of hMSCs in vitro and modulates the hypoxic response through inhibiting HIF-1*α* activity.

Our findings may help to understand the role of SIRT1 in hMSCs in promoting angiogenesis and may contribute to the development of new strategies to improve the hMSCs-based regenerative effects by modulating SIRT1 activity.

## 2. Methods

### 2.1. Reagents

Sirtinol was purchased from Selleck Chemicals LLC (Houston, TX, USA). Culture medium and its supplements including antibiotics and fetal bovine serum (FBS) were purchased from Euroclone (Italy). Primary antibodies against SIRT1 (Abcam, Cambridge, UK), HIF-1*α* (Santa Cruz Biotechnology, Santa Cruz, CA, USA), and tubulin (Sigma-Aldrich, Milan, Italy) were used. Sirtinol was dissolved in dimethyl sulfoxide (DMSO, Sigma-Aldrich) to the appropriate concentrations according to reported procedures. DMSO was also present in the corresponding control.

### 2.2. Cell Lines and Culture Medium

Human mesenchymal stem cells (hMSCs) were obtained from bone marrow as described by Cobellis et al. [8]. Cells were plated in RPMI 1640 growth medium (Euroclone SPA, Italy), containing 10% heat-inactivated FBS, 1% Pen-strep, and 1% L-Glutamine.

Cells were maintained as monolayers in a humidified atmosphere containing 5% CO_2_ at 37°C and the culture medium was replaced every two days.

Hypoxic culture conditions were achieved in a BD GasPak EZ Anaerobe Gas Generating Pouch System (BD Biosciences, San Diego). As certified by the manufacturer, the Anaerobe Gas Generating Pouch System produces an atmosphere containing 10% carbon dioxide and 1% oxygen.

Starvation conditions were obtained incubating cells in RPMI 1640 containing 0.2% FBS.

### 2.3. Infection

After plating, bone marrow cells were grown to confluence and coinfected with HPV16 E6/E7 and hTERT lentiviral vectors (infection number 1). After a week the cells were split and infected again only with hTERT (infection number 2) and cultured until stabilization. Samples were observed and photographed with DMI 6000 inverted microscope (Leica Microsystems) using Leica LAS Image Analysis software (Leica Microsystems). The protocol was also reported in Miceli et al. [27].

### 2.4. hPV16 E6/E7 and hTERT Lentiviral Production

HIV-1based SIN lentiviral vectors were derived from SINF-MU3-W-S vector backbone. hPV16 E6/E7 was inserted upstream of an encephalomyocarditis virus internal ribosome entry site- (IRES-) yellow fluorescent protein (YFP) gene cassette into SINF-MU3-W-S to generate SINF-MU3-E6E7-IRES-YFPW-S. SINF-MU3-hTERT-IRES-GFPW-S was generated by inserting hTERT cDNA upstream of an IRES-green fluorescent protein (GFP) gene cassette into SINF-MU3-W-S. VSV-G-pseudotyped lentiviral vectors were generated in 150 mm tissue culture dishes by transient cotransfection with (1) VSV-G-expressing construct pCMV-VSV-G (Invitrogen, USA) (66 *μ*g), (2) packaging construct pCMVΔR8.2 (addgene) (48 *μ*g), and (3) lentiviral vector plasmids (pSin hTERT or Psin E6-E7) (66 *μ*g) into subconfluent HEK 293FT cells (Invitrogen) by calcium phosphate precipitation (Clontech, Calphos Mammalian Transfection Kit). The supernatant containing the virus was produced in HEK-293FT, collected, filtered, and used to infect bone marrow cells.

### 2.5. Gene Knockdown Using Lentiviral Vector

Cells (10^5^) were grown in RPMI 1640 medium 4.5 g/L glucose (Euroclone SPA, Italy) supplemented with 20% FBS (Euroclone SPA, Italy), 100 U/mL Pen-strep (Lonza Group Ltd), and 2 mM L-Glutamine (Lonza Group Ltd) at 37°C, in 5% CO_2_ fully humidified atmosphere. Cells were first grown for 24 h and then infected with the rLV.H1.sh2Sirt1.EF1.GFP Lentivirus, with a 2.5 MOI, overnight as described by Miceli et al. [27].

### 2.6. RNA Extraction and qPCR

Total RNA was isolated from hMSCs by miRNeasy Mini kit (QIAGEN GE). 500 ng was converted to cDNA using the Quantitect Reverse Transcription kit (QIAGEN GE).

qPCR assays were performed using an iCycler (BioRad Laboratories, USA) and the Sybergreen Super mix (BioRad Laboratories, USA). The primer sequences and qPCR conditions are available on request.

### 2.7. Protein Extraction and Western Blot Analysis

hMSCs were incubated with 100 *μ*M sirtinol for 24 h and then we collected lysates from cells exposed to 1% O_2_ for 6 h.

Cells were lysed in buffer containing 20 mM Tris HCl, 100 mM NaCl, 10 mM MgCl_2_, 1% NP40, 10% glycerol, 0.1 M NaF, 100 *μ*M sodium vanadate, and protease inhibitors mixture (Roche LTD, GE). Equal amounts of supernatant were separated by SDS-polyacrylamide gels. Proteins were transferred to nitrocellulose membranes (WhatmanProtran, GE Healthcare) and membranes were blocked with blocking buffer (TBS-Tween buffer containing 5% milk). Subsequently, the membranes were incubated with primary antibodies at 4°C overnight. After three washes for 10′ with TTBS buffer (50 mM Tris HCl, pH 8, 150 mM NaCl, and 0.5% Tween-20), the membranes were incubated with horseradish peroxidase-conjugated anti-mouse or anti-rabbit antibody (1 : 10.000, Santa Cruz Biotechnology, Santa Cruz, CA, USA) for 1 h at room temperature and then washed for 30 min with TTBS buffer. The resulting immunoblots were detected using Amersham ECL Plus (GE Healthcare).

### 2.8. Senescence Associated Beta-Galactosidase (SA-Beta-Gal) Staining

Cells were cultured on 6-well plates at a density allowing reaching 20–30% confluence and exposed for 24 h to 50 and 100 *μ*M sirtinol. After exposure, the cells were washed three times with inhibitor-free medium and cultured for up to additional 8 days. On the ninth day, the cells were fixed with 2% (v/v) formaldehyde/0.2%  (v/v) glutaraldehyde for 10 min. The cells were then washed twice with PBS and incubated with staining solution (30 mM citric acid/phosphate buffer (pH 6), 5 mM K_4_Fe(CN)_6_, 5 mM K_3_Fe(CN)_6_, 150 mMNaCl, 2 mM MgCl_2_, and 1 mg/mL X-Gal solution (all reagents were purchased from Sigma, Milan, Italy)) at 37°C for 24 h. The cells were photographed and quantified with an inverted microscope (Leica, Heidelberg, Germany).

### 2.9. Cell Proliferation

Cells were plated (5 × 10^3^ cells/well in 96 well plates) in RPMI 1640 (Euroclone SPA, Italy) and allowed to attach overnight. The day after, hMSCs were treated with 100 *μ*M sirtinol for 24, 48, and 72 h. The number of living cells was measured by determination of ATP cellular levels using ViaLight Plus Kit (Lonza Group Ltd). The kit is based upon the bioluminescent measurement of ATP that is present in all metabolically active cells. The bioluminescent method utilizes an enzyme, luciferase, which catalyzes the formation of light from ATP and luciferin. The emitted light intensity is linearly related to the ATP concentration and is measured using a luminometer. RLUs (relative light units) are internal unit of the kit, proportional to the amount of light produced for ATP unit.

All experiments were performed in triplicates.

### 2.10. Flow Cytometry Analysis

Cells were treated with 100 *μ*M sirtinol for 24, 48, and 72 h. Cells were resuspended in the staining solution containing RNAseA, propidium iodide (50 *μ*g/mL), sodium citrate (0.1%), and NP40 (0.1%) in PBS 1X for 30 min in the dark. Cell cycle distribution was assessed with a FACScalibur flow cytometer (Becton Dickinson, San Jose, CA, USA), and 10.000 cells were analyzed by ModFit version 3 Technology (Verity Software House, Topsham, ME, USA) and Cell Quest (Becton Dickinson, San Jose, CA, USA) [27].

### 2.11. Migration Assay

Cells were plated on 24-well plates at a density allowing reaching 50–75% confluence and hMSCs were treated for 24, 48, and 72 h with 100 *μ*M sirtinol. A total of 1.5 × 10^4^ cells were resuspended in 250 *μ*L of RPMI 1640 containing 0.2% FBS and pipetted in the upper chamber of a modified Boyden chamber (Costar Transwell assay, 8 *μ*m pore size, Corning, NY). The chamber was placed in a 24-well culture dish containing 750 *μ*L complete RPMI 1640 with 10% FBS and growth factors. After 24 h incubation at 37°C, transmigrated cells were counted by independent investigators at the inverted microscope.

### 2.12. Capillary Tube Formation Assay in Matrigel

Cells were plated on 6-well plates at a density allowing reaching 50–75% confluence and hMSCs were treated for 24 h with 50 and 100 *μ*M sirtinol. For analysis of capillary tube formation, 150 *μ*L Matrigel (Becton Dickinson, San Jose, CA, USA) was laid into a 96-well plates (BD Falcon, Heidelberg, Germany) and incubated at 37°C for 30 minutes. Cells were trypsinized and 3 × 10^4^ cells were suspended in 150 *μ*L of medium and plated onto Matrigel. Cells were incubated at 37°C and capillary tube formation in Matrigel was observed under an inverted microscope (Leica, Heidelberg, Germany) after 4 and 24 h of incubation.

### 2.13. Statistical Analysis

All data are represented as mean ± S.D. Statistical significance was evaluated by performing Student's *t*-test and significance was accepted if *P* value was <0.05.

## 3. Results

### 3.1. Immortalization of hMSCs

The ectopic expression of hTERT has been reported to extend the life span of cells [28]. However, the use of hTERT alone is not sufficient to immortalize hMSCs, requiring the combinatorial expression of human papillomavirus type 16 genes (HPV16) E6 and E7 [29].

Therefore, primary hMSCs were infected with HPV16 E6-E7 and hTERT lentiviral vectors expressing pSin hTERT and pSin E6-E7 [30] using a multi-infection program, as reported in Methods section. Two clones were obtained and tested for the presence of hTERT and E6-E7 transcripts. Based on RT-PCR data, both clones (MeBM1E1, MeBM1E2) showed similar levels of hTERT and E6-E7 transcripts. No hTERT and E6-E7 expression were detected in untransduced hMSCs (Supplementary Figure  1(a); see the Supplementary Material available online at http://dx.doi.org/10.1155/2014/783459). The resulting cell lines maintained a fibroblast-like phenotype comparable to primary hMSCs and showed no differences in hMSCs markers expression, such as CD73, CD90, and CD105 (Supplementary Figure  1(b)). Thus, these immortalized mesenchymal cells (MeBM1E1, MeBM1E2) represent a valuable model that can be used for basic studies of mesenchymal biology.

### 3.2. Differential Sirtuin Expression in hMSCs

**Figure 1:**
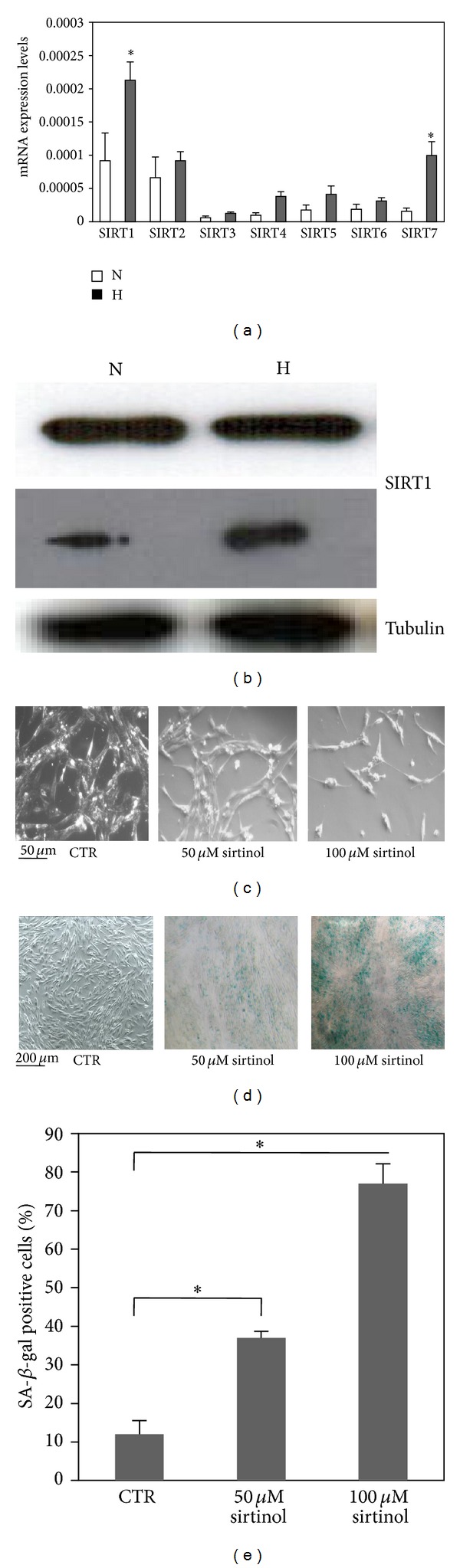
SIRT1–SIRT7 expression in hMSCs and effects of SIRT1 inhibition on phenotype. (a) SIRT1–SIRT7 mRNA levels were measured by real-time PCR (RT-PCR) analyses of total RNAs obtained from hMSCs exposed to either 21% O_2_ (N) or 1% O_2_ (H) for 24 h and relative expression (±SD) were shown. (b) Western blot analysis of hMSCs grown at either 21% O_2_ (N) or 1% O_2_ (H) for 24 h under normal conditions (upper panel) and serum-starved (lower panel). Antibodies against SIRT1 and tubulin were used. (c) Morphological changes in hMSCs were examined 8 days after treatment with sirtinol (50 and 100 *μ*M) for 24 h (40x magnification, scale bars = 50 *μ*m). (d) Representative photographs of blue-stained cells for SA-*β*-Gal activity are shown (10x magnification, scale bars = 200 *μ*m) at 8 days after sirtinol (50 and 100 *μ*M) treatment compared to control (CTR). (e) SA-*β*-Gal-positive cells were quantified by counting in at least 3 random fields for each condition. Results show the mean of three independent experiments. Graph represents means ± SD, *n* = 3. ∗ indicates statistical significance from control, *P* ≤ 0.05.

Using the MeBM1E1 clone, we assessed the expression profile of the Sirt1–Sirt7 genes. We collected RNAs from cells exposed to either 21% O_2_ or 1% O_2_ for 24 h and we performed RT-qPCR analysis to quantify their expression. As shown in Figure 1(a), a significant induction of Sirt1 and Sirt7 (*P* value ≤ 0.05) was detected at mRNA levels in hMSCs under hypoxic conditions compared to normoxia. No significant differences in other sirtuin transcripts were observed under the same conditions.

Next, we evaluated the contribution of hypoxia on SIRT1 protein expression and we collected lysates from cells exposed to 1% O_2_ for 24 h in the presence or absence of growth factors (i.e., serum).

As shown in Figure 1(b), Western blot analysis showed that there was no change in SIRT1 protein levels in hMSCs exposed to 1% O_2_ for 24 h grown in presence of serum, whereas hypoxia increased SIRT1 levels when hMSCs were cultured in low serum conditions. These data showed that hypoxia alone did not stimulate SIRT1 protein accumulation, whereas depletion of growth factors in combination with hypoxia resulted in an increase of SIRT1 protein expression.

### 3.3. Inhibition of SIRT1 Induces Premature Senescence-Like Phenotype in hMSCs

To evaluate the effects of targeting SIRT1, we decided to use pharmacological and genetic approaches to inhibit SIRT1. Genetic inhibition was obtained by silencing SIRT1 with lentiviral vector expressing sh-Sirt1-GFP. In order to determine the infection efficiency, green fluorescent protein (GFP) was monitored using fluorescence microscopy after 10 days from infection. As shown in Supplementary Figure  2(a), high levels of GFP in sh-Sirt1-hMSCs were observed with a concomitant reduction of SIRT1 protein (Supplementary Figure  2(b)). In addition, pharmacological inhibition was obtained by sirtinol, a cell permeable specific inhibitor of SIRT deacetylase activity [19].

To investigate whether SIRT1 modulates premature senescence-like phenotype in hMSCs, we examined the effect of SIRT1 inhibition in our cells. hMSCs were treated with sirtinol at 50 and 100 *μ*M for 24 h. After exposure, the cells were washed with inhibitor-free medium and cultured for additional 8 days. As shown in Figure 1(c), sirtinol induced senescence-like morphological changes in hMSCs that showed enlarged and flattened shapes with a concomitant cell number reduction. Then, we evaluated SA-*β*-gal activity, a characteristic feature of senescence phenotype. Sirtinol increased SA-*β*-gal activity in hMSCs compared to control. Importantly, sirtinol increased SA-*β*-gal activity in a dose-dependent manner (Figures 1(d)-1(e)). To confirm the prosenescence role of SIRT1 inhibition, we examined SA-*β*-gal activity in sh-Sirt1 infected cells. As expected, similar results were obtained in sh-Sirt1 cells (data not shown). These results demonstrated that inhibition of SIRT1 induced a senescence phenotype in hMSCs.

### 3.4. Effects of SIRT1 Inhibition on Proliferation

**Figure 2:**
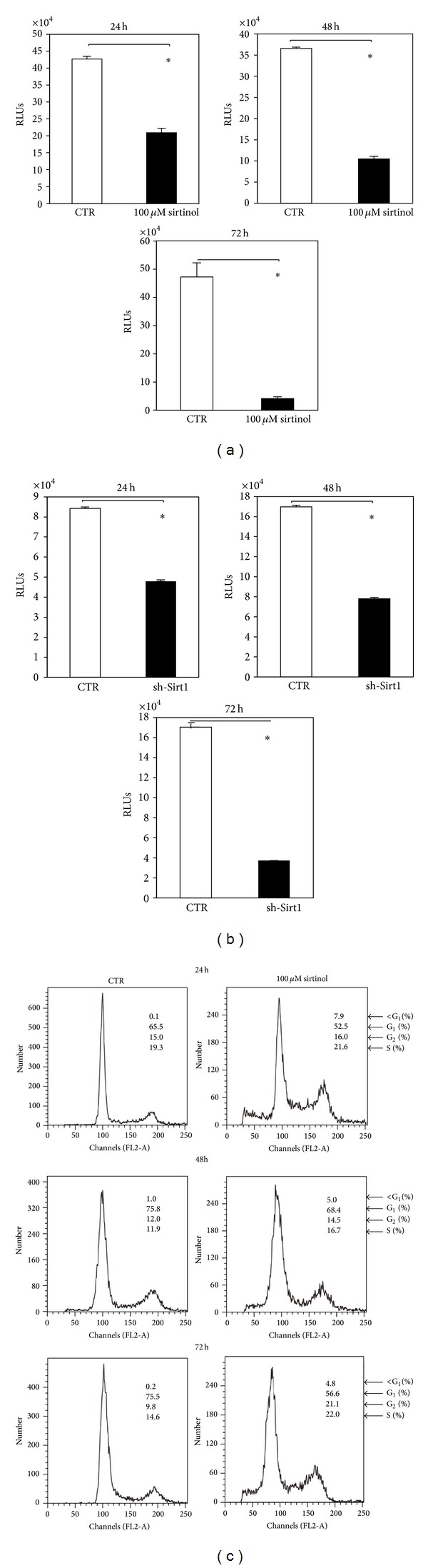
Effects of SIRT1 inhibition on proliferation, cell cycle, and apoptosis. (a) hMSCs were treated with sirtinol 100 *μ*M (black bars) or equivalent concentration of DMSO (white bars) for 24, 48, and 72 h. (b) sh-Sirt1 infected hMSCs were plated and proliferation was measured for 24, 48, and 72 h. Each histogram indicates the RLUs^#^ related to cell growth measured at different times. Error bars represent SD of *n* = 3. ∗ denotes statistical differences when *P* ≤ 0.05 is compared to control. (c) hMSCs were treated with sirtinol 100 *μ*M or equivalent concentration of DMSO for 24, 48, and 72 h. The cells stained with propidium iodide (PI) were subjected to flow cytometric analysis to determine the cell distributions at each phase of the cell cycle. ^#^RLUs are relative light units (RLUs) related to ATP cellular level.

We then examined the effects of SIRT1 inhibition on the proliferation of hMSCs. Cells were treated as previously described and proliferation was measured by intracellular levels of ATP at different times (24, 48, and 72 h). Sirtinol significantly inhibited the proliferation of hMSCs in a time-dependent manner compared to control. Similar results were obtained in sh-Sirt1 hMSCs (Figures 2(a)-2(b)).

To investigate whether inhibition of SIRT1 induced growth arrest or cell death, a cell cycle analysis was performed on hMSCs treated with sirtinol for 24, 48, and 72 h by fluorescence activated cell sorting (FACS). By this analysis, we observed a significant cell accumulation in pre-G1 phase, corresponding to apoptotic cells after 24, 48, and 72 h of treatment compared to untreated cells. No significant effect was observed in G1, G2, and S phase (Figure 2(c)), compared to control. Similar results were obtained in sh-Sirt1 cells (data not shown). These data suggested that inhibition of SIRT1, obtained by both pharmacological and genetic approaches, induced apoptosis in hMSCs, without altering the cell cycle distribution of the cells.

### 3.5. SIRT1 Inhibition Impairs Migration and Capillary Tube Network Formation

**Figure 3:**
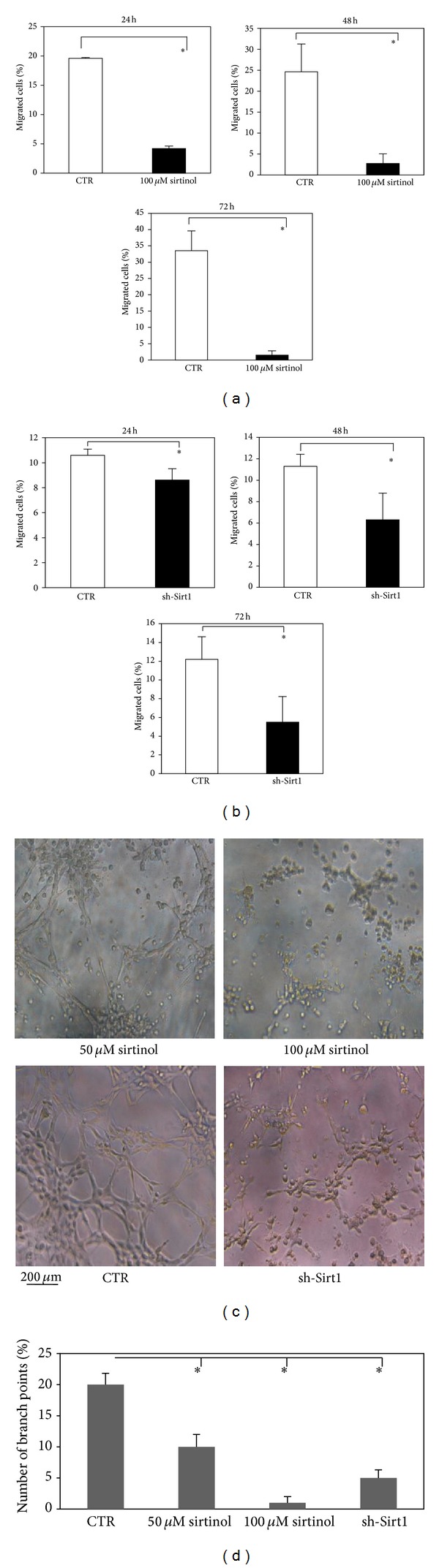
Effects of SIRT1 inhibition on migration and capillary tube network formation. (a) hMSCs were treated with sirtinol 100 *μ*M (black bars) or equivalent concentration of DMSO (white bars) for 24, 48, and 72 h. (b) sh-Sirt1-hMSCs migration (black bars) compared to control (white bars) at 24, 48, and 72 h. Each histogram indicates the % of migrated cells measured at different times. Statistical differences were denoted with ∗ when *P* ≤ 0.05 is compared to control. (c) Tubule formation promoted by hMSCs treated with sirtinol (50 and 100 *μ*M) (upper panel) and tubular structures of sh-Sirt1 infected hMSCs plated on Matrigel compared to control (lower panel). Magnification 10x. Scale bar = 200 *μ*m. (d) Tubule formation was quantified by counting the number of branch points of the capillary network. Data are expressed as mean ± SD. ∗ denotes statistical differences when *P* ≤ 0.05 is compared to control.

Then, we examined whether inhibition of SIRT1 impinged on the migration ability of hMSCs. Cells were exposed for 24, 48, and 72 h to 100 *μ*M sirtinol and we examined the migratory ability of hMSCs in presence of growth factors. The number of migrated cells was significantly reduced in cells treated with SIRT1 inhibitor in a time-dependent manner (Figure 3(a)), compared to control. Consistent with these findings, silenced SIRT1 resulted in a significant reduction of migration compared to control (Figure 3(b)).

These results suggested that inhibition of SIRT1 reduces the capability of hMSCs to migrate. To further assess whether inhibition of SIRT1 might play a role in the ability to form capillary-like networks, we performed a tubule formation assay. We used Matrigel as the basement matrix to induce tubule formation. Cells were exposed to sirtinol (50 and 100 *μ*M) for 24 h; then sirtinol was removed from the culture media. The cells were plated on Matrigel and allowed to form tubule networks in vitro. As shown in Figure 3(c), we found that hMSCs exposed to 50 *μ*M sirtinol formed less developed tubule structures than untreated cells within a 4-hour period and the number of branch points significantly diminished (Figure 3(d)). Additionally, cell treatment at a concentration of 100 *μ*M sirtinol resulted in the complete suppression of tubule-like structure formation, in contrast to stable tubular networks present in the control (Figure 3(c), upper panel-3D). A reduced ability to form tubule-like formation was also observed in sh-Sirt1 cells compared to control (Figure 3(c) lower panel-3D). These data indicated that SIRT1 activity is involved in tubule-like formation of hMSCs.

### 3.6. Impaired Angiogenic Properties of hMSCs Resulting from SIRT1 Inhibition Are Mediated by HIF-1*α* Protein

To investigate whether SIRT1 activity could regulate HIF-1*α* accumulation, we analyzed the effects of SIRT1 inhibition on HIF-1*α*.

**Figure 4:**
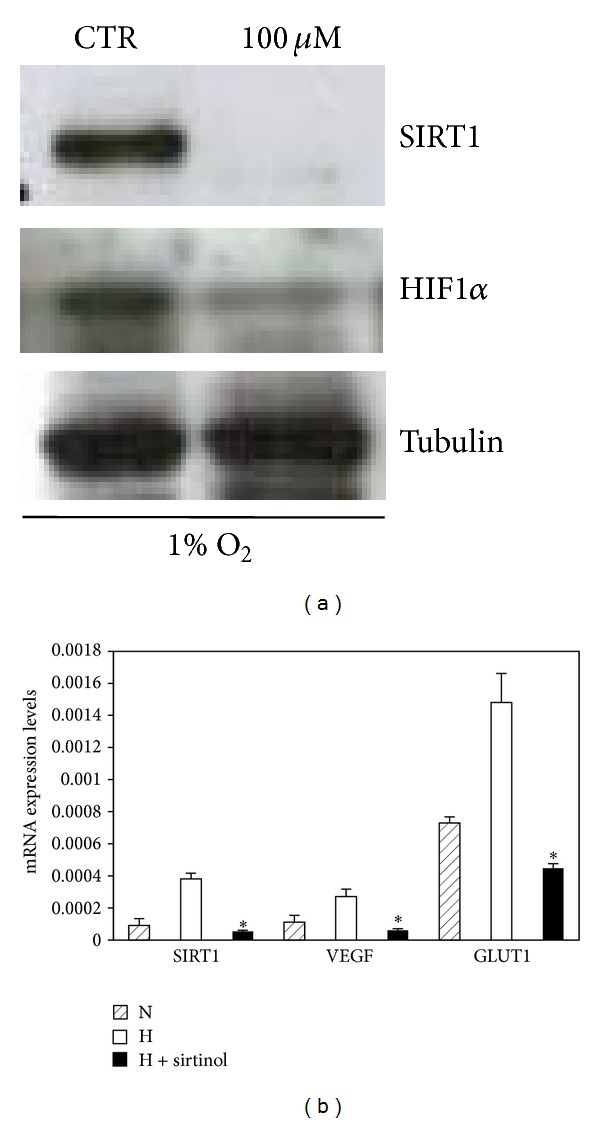
Effects of sirtinol on SIRT1 expression and HIF-1*α*. (a) hMSCs were treated with sirtinol (100 *μ*M) or equivalent concentration of DMSO (CTR) for 24 h and then exposed to 1% O_2_ for 6 h. All cells lysates were analyzed for SIRT1, HIF-1*α*, and tubulin by Western blot. (b) The amounts of SIRT1, VEGF, and GLUT1 mRNA of hMSCs treated with sirtinol at concentration of 100 *μ*M (black bars) or equivalent concentration of DMSO (white bars) for 24 h and exposed to 1% O_2_ (H) for 6 h were quantified with real-time PCR. The levels of SIRT1, VEGF, and GLUT1 mRNA of hMSCs exposed to 21% O_2_ (N) for 24 h were measured. ∗ indicates statistical significance from control, *P* ≤ 0.05.

Cells were incubated with 100 *μ*M sirtinol for 24 h and then exposed to 1% O_2_ for 6 h, known to induce HIF-1*α*. We then collected cell lysates and Western blotting analysis was performed. Interestingly, as shown in Figure 4(a), inhibition of SIRT1 led to a reduction of SIRT1 protein, as expected, and a strong repression of HIF-1*α* protein accumulation in hypoxic conditions, compared to control.

To determine if reduction in HIF-1*α* levels, conferred by SIRT1 inhibition, could affect HIF-1*α* transcriptional activity, we looked at the expression of known HIF-1*α* target genes. Cells were treated with 100 *μ*M sirtinol for 24 h and exposed to either 21% O_2_ or 1% O_2_ for 6 h. Then, we collected RNAs from cells and RT-qPCR analysis was performed. As shown in Figure 4(b), the treatment with sirtinol in combination with hypoxia significantly reduced the hypoxic induction of SIRT1 expression and HIF-1*α* target genes: Glut1 and VEGF. These data indicated that inhibition of SIRT1 decreased HIF-1*α* protein accumulation and its transcriptional activity under hypoxic conditions.

## 4. Discussion

In the present study, we have generated an immortalized human bone marrow-derived mesenchymal cell line (MeBM) that represent a valuable tool to study mesenchymal biology and we investigated whether SIRT1 can influence angiogenic capacity of these cells.

Cell therapy with hMSCs is a promising and safe modality with the potential for vascular regeneration in the treatment of several diseases, especially critical limb ischemia. Pilot human studies have shown that autologous transplantation of bone marrow cells induced therapeutic angiogenesis in patients with critical limb ischemia [3]. hMSCs are pluripotent progenitors that can differentiate into a variety of cell types and have been shown to promote angiogenesis both in vivo and in vitro. It is widely accepted that hMSCs present in bone marrow are therapeutic cells. Emerging evidence suggests that most of the beneficial effects of hMSCs can be explained by the secretion of soluble factors that induce endogenous reparatory processes. However, the mechanisms by which hMSCs promote angiogenesis are not clear.

The present investigation was undertaken to identify the function, if any, of SIRT1 in hMSCs and clarify the molecular mechanism by which SIRT1 regulate angiogenesis in hypoxic conditions.

Sirtuins represent a family of NAD+-dependent protein deacetylases involved in several pathologies. In mammals, SIRT1 is the most closely related homologue of yeast Sir2 and belongs to class III histone deacetylases. It regulates a wide variety of biological functions including gene expression, cell survival, proliferation, differentiation, metabolism, immune response, carcinogenesis, and angiogenic response through multiple targets [15].

Investigation of sirtuin expression was an important preliminary step to study the selective role of class III HDACs. Since sirtuins have been shown to respond to perturbations in the ratio of oxidized NAD+/reduced NADH and, therefore, to modulate the response to hypoxic stress [31], we first looked at sirtuin expression of the family members, both in normoxic and hypoxic conditions.

Therefore, we assessed the expression profile of the Sirt1–7 genes in our cells. We found that SIRT1 and SIRT7 were significantly upregulated in hypoxic conditions compared to normoxia.

SIRT1 activation improves endothelial function and suppresses vascular inflammation, two central pathophysiological processes involved in the initiation and progression of cardiovascular disease [32]. In particular, SIRT1 has been shown to protect endothelial cells from premature senescence and to regulate angiogenesis and vascular tone [26]; thus we decided to focus our study on SIRT1.

In our study, we evaluated the contribution of hypoxia on SIRT1 protein, showing that no change in SIRT1 was detected in hMSCs grown in presence of growth factors, whereas SIRT1 protein expression increased in hMSCs grown in absence of growth factors under hypoxic conditions. Other experiments will be necessary to clarify this; however, our findings might indicate that SIRT1 may act as a sensor of the metabolic state of MSCs in stress conditions (i.e., hypoxia and absence of growth factors).

Several studies have demonstrated that SIRT1 prevents the onset of senescence in multiple cell types, which exhibit alterations in morphology and gene expression that extinguish essential cellular functions [31].

It is known that primary cells underwent senescence when cultured in vitro, showing increased activity of *β*-galactosidase (*β*-gal) when assayed at pH 6 [22]. Therefore, we decided to evaluate whether inhibition of SIRT1 might play a role in undertaking a senescent phenotype in our cells. We found that sirtinol, a cell-permeable 2-hydroxy-1-napthaldehyde derivative that acts as a specific and direct inhibitor of all NAD+-dependent protein deacetylases of sirtuin family, significantly increased SA-*β*-gal activity in a concentration-dependent manner and sustained enlarged and flattened cell morphology.

Our results are consistent with previous studies in human endothelial cells [22, 33], mouse fibroblasts [34], and human cancer cells [35], concluding that sirtuins are implicated in cellular senescence.

It has been demonstrated that senescence alters mesenchymal stem cell properties, such as proliferation and migration [36] and activity of SIRT1 has been linked to this condition [37]. In fact, when cells undergo senescence, their proliferation declines significantly.

Our findings showed that SIRT1 inhibition significantly inhibited the proliferation rate of hMSCs in a time-dependent manner and significantly induced apoptosis in hMSCs.

Going through our data, we also noticed that the effect of genetic inhibition of SIRT1 on angiogenesis response of hMSCs was less pronounced than pharmacological modulation of SIRT1. A possible explanation for this observation is that sirtinol can inhibit other NAD+-dependent protein deacetylases of sirtuin family, especially SIRT2 [22]. In conclusion, we suggested that inhibition of SIRT1 both by pharmacological and genetic approaches reduces significantly angiogenic properties in cultured hMSCs.

The connection between sirtuin and HIF proteins is complex and the current literature is in part contradictory [19]. Recent reports have linked HIF to sirtuin families by demonstrating that SIRT1, SIRT3, SIRT6, and SIRT7 can regulate the activity of HIF proteins [19, 38]. Lim et al. [18] demonstrated that SIRT1 binds to and deacetylates HIF-1*α* at lysine 674. This interaction blocks p300 recruitment to the promoter of HIF-1*α* target genes and thereby represses HIF-1*α* transcriptional activity, whereas Laemmle et al. reported that SIRT1 increased HIF-1*α* protein levels [19].

In contrast, Dioum et al. [17] have reported that SIRT1 does not target HIF-1*α*; it rather deacetylates HIF-2*α* and their interaction promotes HIF-2*α* transcriptional activity. In addition, because SIRT1 is a redox cellular sensor and dependent on metabolic status of the cell, its regulation by hypoxia has been a point of interest. In one report, SIRT1 is downregulated in hypoxic conditions due to decreased NAD+ levels [18], while in another study it is upregulated in a HIF-dependent manner [39].

Thus, the interaction between SIRT1 and HIF factors and the resulting outcome of their interactions are still unclear. In this study we found that HIF-1*α* transcriptional activity is impaired by SIRT1 inhibition under hypoxic conditions, as reported in [19].

## 5. Conclusions

Our results suggest that SIRT1 exert a role in angiogenic properties of hMSCs. Thus, our study might have important implications in the field of angiogenesis, possibly leading to the identification of chemical compounds that can positively regulate SIRT1, improving regenerative processes exerted by hMSCs.

## Supplementary Material

Supplementary Figure 1
Primary hMSCs were infected with HPV16 E6-E7 and hTERT lentiviral vectors expressing pSin hTERT and pSin E6-E7 [30] using a multi-infection program, as reported in Methods section. Two clones were obtained and tested for the presence of hTERT and E6-E7 transcripts. Based on RT-PCR data, both clones (MeBM1E1, MeBM1E2) showed similar levels of hTERT and E6-E7 transcripts. No hTERT and E6-E7 expression were detected in untransduced hMSCs (Supplementary Figure 1(a)). The resulting cell lines maintained a fibroblast-like phenotype comparable to primary hMSCs and showed no differences in hMSCs markers expression, such as CD73, CD90, and CD105 (Supplementary Figure 1(b)).Thus, these immortalizedmesenchymal cells (MeBM1E1, MeBM1E2) represent a valuable model that can be used for basic studies of mesenchymal biology.
Supplementary Figure 1 (Legend): A) RT-PCR analysis was performed on MeBM1E1 and MeBM1E2 clones to detect expression of hTERT and E6-E7 transcripts and (1B) different hMSCs markers expression compared to untransducted cells. M= molecular weight marker
Supplementary Figure 2
Genetic inhibition was obtained by silencing SIRT1 with lentiviral vector expressing sh-Sirt1-GFP. In order to determine the infection efficiency, green fluorescent protein (GFP) was monitored using fluorescence microscopy after 10 days from infection. As shown in Supplementary Figure 2(a), high levels of GFP in sh-Sirt1- hMSCs were observed with a concomitant reduction of SIRT1 protein (Supplementary Figure 2(b)).
Supplementary Figure 2 (Legend): A) Representative picture of high levels of GFP in sh-Sirt1-hMSCs (40x magnification, Scale bars=50 *µ*m). B) SIRT1 and tubulin were analyzed by Western blotting in hMSCs and sh- Sirt1-hMSCs.
