# Supplementary figures and images for: SIRT1 Inhibition Affects Angiogenic Properties of Human MSCs
Source: Biomed Res Int. 2014 Aug 27;2014:783459. doi: 10.1155/2014/783459 (PMC4163475; doi:10.1155/2014/783459)

A

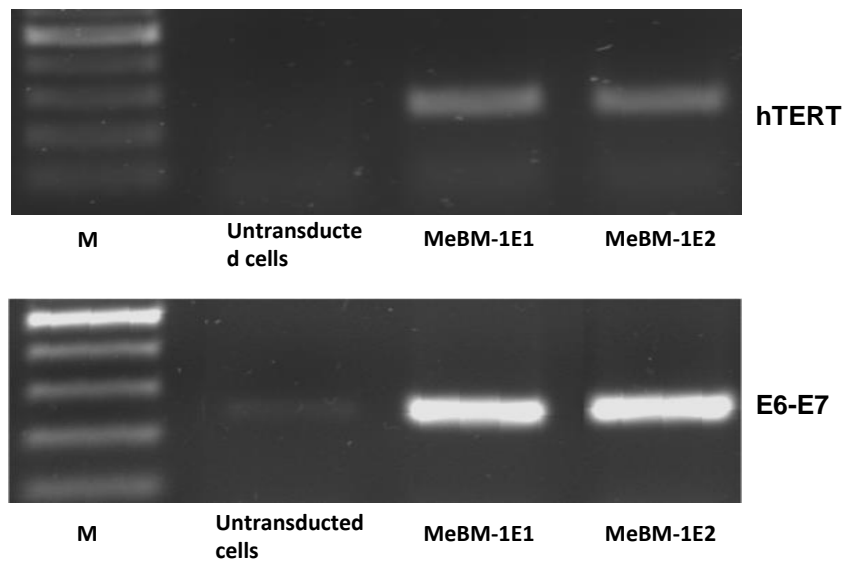

B

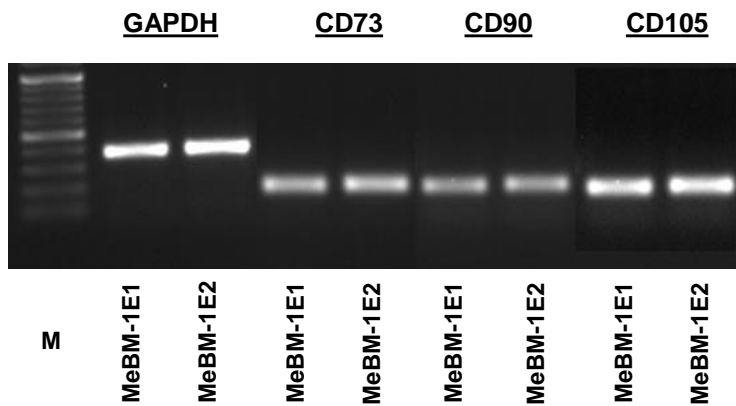

A

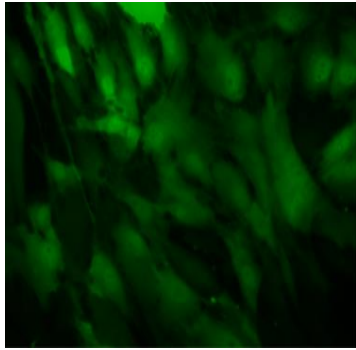

50  $\mu$ m

shsirt1

B

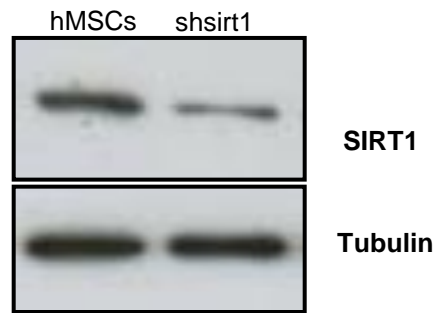

Supplement: Supplementary file 1 — Supplementary Figure 1 Primary hMSCs were infected with HPV16 E6-E7 and hTERT lentiviral vectors expressing pSin hTERT and pSin E6-E7 [30] using a multi-infection program, as reported in Methods section. Two clones were obtained and tested for the presence of hTERT and E6-E7 transcripts. Based on RT-PCR data, both clones (MeBM1E1, MeBM1E2) showed similar levels of hTERT and E6-E7 transcripts. No hTERT and E6-E7 expression were detected in untransduced hMSCs (Supplementary Figure 1(a)). The resulting cell lines maintained a fibroblast-like phenotype comparable to primary hMSCs and showed no differences in hMSCs markers expression, such as CD73, CD90, and CD105 (Supplementary Figure 1(b)).Thus, these immortalizedmesenchymal cells (MeBM1E1, MeBM1E2) represent a valuable model that can be used for basic studies of mesenchymal biology. Supplementary Figure 1 (Legend): A) RT-PCR analysis was performed on MeBM1E1 and MeBM1E2 clones to detect expression of hTERT and E6-E7 transcripts and (1B) different hMSCs markers expression compared to untransducted cells. M= molecular weight marker Supplementary Figure 2 Genetic inhibition was obtained by silencing SIRT1 with lentiviral vector expressing sh-Sirt1-GFP. In order to determine the infection efficiency, green fluorescent protein (GFP) was monitored using fluorescence microscopy after 10 days from infection. As shown in Supplementary Figure 2(a), high levels of GFP in sh-Sirt1- hMSCs were observed with a concomitant reduction of SIRT1 protein (Supplementary Figure 2(b)). Supplementary Figure 2 (Legend): A) Representative picture of high levels of GFP in sh-Sirt1-hMSCs (40x magnification, Scale bars=50 µm). B) SIRT1 and tubulin were analyzed by Western blotting in hMSCs and sh- Sirt1-hMSCs. [file 783459.f1.pdf]
